# Use of online food delivery services among adults in five countries from the International Food Policy Study 2018–2021

**DOI:** 10.1016/j.pmedr.2024.102766

**Published:** 2024-05-22

**Authors:** Adyya Gupta, Gary Sacks, Adrian J. Cameron, Catherine E. Huggins, Anna Peeters, Kathryn Backholer, Lana Vanderlee, Christine M. White, Tailane Scapin, Clara Gomez-Donoso, Rebecca Bennett, Joel A Dubin, David Hammond

**Affiliations:** a Deakin University, Geelong, Australia, Institute for Health Transformation, Global Centre for Preventive Health and Nutrition, School of Health and Social Development, Faculty of Health, VIC 3220, Australia; b School of Nutrition, Centre NUTRISS (Nutrition, santé et société), Institute of Nutrition and Functional Foods, Université Laval, Quebec, QC G1V 0A6, Canada; c School of Public Health Sciences, Faculty of Health, University of Waterloo, Waterloo, ON N2L 3G1, Canada; d Department of Statistics and Actuarial Science, University of Waterloo, Waterloo, ON N2L 3G1, Canada

**Keywords:** Online food delivery, Food environment, Diet, Non-communicable diseases, Public health, Food delivery, Trends

## Abstract

**Aim:**

Online food delivery services (OFDS) are popular for purchasing meals prepared outside home, increasing access to energy-dense and nutrient-poor foods. This adversely impacts dietary choices and health outcomes. Our study examined trends in OFDS use in Australia, Canada, Mexico, the United Kingdom (UK), and the United States (US) from 2018 to 2021.

**Methods:**

Repeated annual cross-sectional data was sourced from the International Food Policy Study for five countries among adults over 18 years (N = 83,337). Weighted estimates for trends in i) the proportion of the respondent’s purchasing meals per week using OFDS, and ii) average number (and standard deviation (SD)) of meals purchased per week using OFDS were assessed. Logistic regression models were fitted.

**Findings:**

OFDS use increased among adults between 2018–2021 (Australia: 17 % of respondents purchased at least one meal in the last 7 days using OFDS in 2018 to 25 % in 2021, Canada: 12 % to 19 %, Mexico: 28 % to 38 %, UK: 19 % to 28 %, and US: 17 % to 21 %). Average number of meals purchased per week outside home remained consistent for all countries over time (e.g., in Australia, 2.70 (SD 0.06) meals in 2018 and 2.63 (SD 0.06) in 2021). However, average number of meals purchased using OFDS nearly doubled between 2018 and 2021 (e.g., in Australia, 0.45 (SD 0.03) meals in 2018 to 0.81 (SD 0.04) in 2021).

**Conclusion:**

OFDS use is increasing and are substituting the conventional forms of purchasing meals outside home. Nutritional quality of foods sold, marketing practices and purchasing patterns on OFDS deserve further attention.

## 1 Background

Diets high in energy-dense and nutrient poor foods (i.e. foods containing high amounts of added sugars, salt and saturated fat) are a key risk factor for non-communicable diseases, including obesity (World Health Organisation, 2021). Retail food environments play an important role in determining population diets and health outcomes (Peeters, 2018). Emerging evidence shows that foods prepared outside home, and purchased from retailers such as fast food and takeaway food outlets are often nutrient poor and their regular consumption has been associated with high daily energy intakes and an increased risk of obesity (Gopinath et al., 2016, Smith et al., 2009, Jaworowska et al., 2014, Lachat et al., 2012, Nago et al., 2014).

Digitalization of the food environment has enabled new ways to buy and sell food, with online food delivery services (OFDS) experiencing substantial growth in the last two decades (Morgan, 2020). OFDS, such as Uber Eats® (Australia), GrubHub® (United States (US)), Just Eat® (United Kingdom (UK)) and SkiptheDishes (Canada), provide an avenue for food ordering and delivery from a range of retailers over a large geographic area on a single platform, and incorporate the convenience of rapid, trackable home delivery (Brar and Minaker, 2021). OFDS is becoming a popular mode of purchasing meals prepared outside home, with a forecasted global increase from 0.8 billion users in 2018 to almost 2.9 billion users in 2029 (Statistica, 2023). Factors that have contributed to the growth in OFDS use include increased access to the internet via mobile phones, increased sophistication of apps, and greater reliance on home delivery of food during the Coronavirus disease 2019 (COVID-19) pandemic (Morgan, 2020, Nielson, 2015).

Limited evidence from Canada, the US, the Netherlands, Australia, and New Zealand shows that the majority of the foods available on OFDS are nutrient poor (Brar and Minaker, 2021, Mahawar et al., 2022, Partridge et al., 2020, Poelman et al., 2020). For example, 73% (18,955/25,877) (Mahawar et al., 2022) and 86% (4958/5769) (Partridge et al., 2020) of the menu items assessed on two widely used OFDS in New Zealand and Australia, respectively, were classified as discretionary foods (energy dense and nutrient poor foods), according to the Australian Dietary Guidelines, including items such as burgers and pizzas. In addition to being dominated by unhealthy foods, OFDS make foods prepared outside home readily available and accessible. For example, an OFDS widely used in Ontario, Canada was found to offer access to foods from 472 restaurants from up to 9 km radius (Brar and Minaker, 2021).

Recent data from Australia (Statistica, 2019), the US (Fryar et al., 2018) and the UK (Adams et al., 2015) suggests that overall consumption of food prepared outside the home has increased, with over a third of the population purchasing at least one meal per week that was prepared outside home in these countries. Food prepared outside home was traditionally acquired either in-person from a food outlet or ordering directly from a restaurant to then pick up in-person (Cameron et al., 2022). With emerging demand for convenience (Bates et al., 2023), there has been an upsurge in the popularity of OFDS (Statistica, 2023). There is only limited evidence, from 2018, of socio-demographic characteristics of OFDS users across Australia, Canada, Mexico, the UK, and the USA (Keeble et al., 2020). The extent to which food purchases using OFDS, compared to those using in-person food purchasing, affects the overall quantity of consumption of foods prepared outside home, and the healthiness of dietary intake overall, remains unknown.

To inform and advocate for actions that ensure that OFDS do not result in an increased risk of unhealthy diets and related health outcomes, it is important to understand trends in the use of OFDS relative to other conventional ways of purchasing food prepared outside home. This study aimed to examine trends in OFDS use for purchasing meals in Australia, Canada, Mexico, the UK, and the US from 2018 to 2021.

## 2 Methods

### 2.1 Study Design

Annual cross-sectional data from 2018 to 2021 was sourced from the International Food Policy Study (IFPS), conducted in Australia, Canada, Mexico, the UK, and the US. The data from 2018 to 2021 were selected because the data collected in the prior wave (2017) did not assess meals ordered using OFDS. Self-administered web-based surveys were conducted annually in November-December with adult respondents aged 18 to 100 years recruited through the Nielsen Consumer Insights Global Panel and their partners’ panels. Email invitations with unique survey access links were sent to a random sample of panelists in each country. Consent was obtained from the participants prior to commencing the survey; participants were incentivized using their panel’s existing reward structure. Further detail on the study methodology can be found in the IFPS Technical Reports (Hammond et al., 2021). The IFPS was reviewed by and received ethics clearance through a University of Waterloo Research Ethics Committee (ORE#30829). Further ethics clearance for this secondary data analysis was obtained from the Deakin University Research Ethics Committee (DUHREC#2023–100).

### 2.2 Measures

Meals prepared outside home were measured through the following question: “During the past 7 days, how many meals did you get that were prepared outside home in places such as restaurants, fast food or takeaway places, food stands, or from vending machines?”. Respondents could enter number of meals ranging from 0 to 21. Next, respondents who had purchased at least one meal prepared outside home reported the number of these meals that were: a) ordered using an OFDS (e.g., Uber Eats® and other country-specific examples), b) ordered directly from a restaurant and delivered, c) purchased in person at a restaurant/food outlet within 5 minutes of their home, excluding delivery, and d) purchased in person at a restaurant/food outlet more than 5 minutes from their home, excluding delivery. Respondents could also select “Don’t know” or “Refuse to answer” for both outcomes. OFDS use was assessed through this follow up question.

Sociodemographic characteristics included were based on existing literature relating to purchasing meals from OFDS (Keeble et al., 2020, Keeble et al., 2021). Key variables of interest included age group (18–29, 30–44, 45–59 or 60–100 years), sex at birth (male or female), and living with children aged under 18 years (yes or no). Ethnicity was assessed using country-specific race/ethnicity categories and analysed as a derived variable to accommodate different measures across countries (‘majority’: white, predominantly English speaking or not indigenous; or ‘minority’: all other responses). Education level was categorised as ‘low’ (high school completion or lower), ‘medium’ (some post-high school qualifications), or ‘high’ (university degree or higher), and was used as a proxy for socioeconomic status (Galobardes et al., 2001).

### 2.3 Statistical analysis

To ensure that the study data closely resemble the population socio-demographics in all countries, data were weighted with post-stratification sample weights constructed using population estimates sourced from the census estimates in each country based on age group, sex, region, ethnicity, and education. The methodology employed for post-stratification is described elsewhere (Hammond et al., 2022). Due to the differences in sampling and weighting in the data, oversample (comprising of respondents with low educational attainment from Mexico and Mexican Americans in the US for 2021, n = 5147) and respondents with missing data (“refuse to answer” and “don’t know” responses for variables of interest, including ethnicity, education and living with children aged under 18 years from 2018 to 2021 (n = 1243)) were excluded from the final analytical sample. Similar weights were applied across 2018 to 2021 data for consistency. Descriptive statistics were used to summarise the weighted estimates of the sociodemographic characteristics of the total sample across 2018–2021. Respondent purchasing food outside home was categorised into two categories (as a binary outcome): a) meals prepared exclusively at home and b) purchased at least one meal prepared outside home (either using or not using OFDS). Respondents’ OFDS use was categorised into three categories (as a multinomial outcome): as: a) meals prepared exclusively at home, b) purchased at least one meal prepared outside home not using OFDS, and c) purchased at least one meal prepared outside home using OFDS. The derived OFDS use variable was used to estimate the annual proportion (and 95 % confidence interval (CI)) of the respondents purchasing meals prepared outside home using OFDS in the past 7 days, by country and year. Proportions for categorical variables were described by frequencies and percentages.

To investigate trends over time, two regression models were fitted, one multiple logistic regression model on the binary outcome (purchasing food outside home) and one multiple multinomial logistic regression model on the multinomial outcome (purchasing food outside home using OFDS), with ‘food prepared exclusively at home’ as the reference category in both the models. An indicator for country was included in the main model in which all countries were combined. All models were adjusted for sociodemographic variables including age, sex, ethnicity, education and living with children aged under 18 years. Year of survey was fitted as a categorical covariate with 2018 as the base year and its coefficients represented changes in odds of the respective outcome over time. Models were run for all countries combined and for each country separately.

For each year (hereon referred to as annually) and country, average number (and standard deviation (SD)) of meals prepared outside home in the past 7 days was estimated based on purchases from OFDS and other purchase formats including deliveries ordered directly from a restaurant, and in-person purchases at a restaurant/food outlet within or more than 5 minutes of home (excluding delivery). The denominator included all participants (e.g., who did and did not report purchasing food prepared outside home). All analysis was conducted using Stata SE 17 version.

## 3 Results

Table 1 describes the annual distribution of the weighted sample characteristics from 2018 to 2021 across the five countries. The final analytical sample included 83,337 respondents. Overall, across five countries, between 2018 and 2021, the mean (±SD) age of the sample was 46.1 ± 0.07 years. The total sample was comprised of nearly equal proportion of males and females, 78 % were of ethnic majority, nearly 57 % had medium or high education, and approximately 30 % lived with children aged under 18 years.

**Table 1:** Analytic sample characteristics (weighted), International Food Policy Study, 2018–2021 (N = 88337).

	Total sample (N = 88337)	2018 (n = 21986)		2019 (n = 20211)		2020 (n = 20905)		2021 (n = 20235)	
Variables	Categories	n	(%)	n	(%)	n	(%)	n	(%)
Country	Canada	4169	18.9	3298	19.4	4125	19.7	4364	20.7
	Australia	3979	18.1	4078	20.2	4136	19.8	3947	18.8
	UK	5335	24.2	3978	19.7	4097	19.6	3983	18.9
	US	4495	20.4	4047	19.9	4443	21.3	3913	29.3
	Mexico	4008	18.4	4180	20.7	4104	19.7	4028	12.3

Age group	18–29 years	4594	22.1	4292	22.0	4459	21.4	4105	20.1
	30–44 years	5195	26.2	5461	26.7	5486	26.3	5195	25.0
	45–59 years	5216	26.1	5103	25.7	5356	25.8	4896	25.2
	60–100 years	6261	25.6	5355	25.6	5604	26.5	6039	29.6

Sex at birth	Male	10,797	48.7	10,004	48.7	10,318	48.7	10,014	49.3
	Female	11,189	51.4	10,207	51.3	10,587	51.3	10,221	50.7

Ethnicity	Majority	18,385	78.0	16,676	77.5	16,669	77.1	16,562	78.8
	Minority	3601	22.0	3535	22.5	4236	22.9	3673	21.2

Education	Low	5906	42.6	5858	42.7	7147	42.8	5868	47.9
	Medium	5998	22.3	5467	21.8	5690	21.8	5457	21.5
	High	10,082	35.1	8886	35.5	8068	35.4	8910	30.6

Living with children < 18 years	No	15,404	70.3	13,851	68.8	14,202	69.1	13,936	71.8
	Yes	6581	29.7	6360	31.2	6703	30.9	6299	28.2

### 3.1 Annual proportion of the respondents purchasing meals prepared outside home in the past 7 days

Table 2 describes weighted proportions of respondents purchasing at least one meal prepared outside home (either using or not using OFDS) in the last 7 days across countries between 2018–2021. In 2018, 80 % of participants across countries purchased at least one meal prepared outside home in the last 7 days. A decline in the proportion of respondents was observed for all countries in 2020, where 73 % of respondents had purchased at least one meal prepared outside home in the last 7 days, but this increased to 76 % in 2021 across all five countries. In the multiple binomial logistic regression model, both the unadjusted and adjusted models, compared to 2018, lower odds of purchasing meals prepared outside home (either or not using OFDS) were observed in 2020 and 2021 for all countries combined as well as across each country (Table 3). These trends were statistically significant (p < 0.001) in all countries combined and country-specific unadjusted and adjusted models, except for Australia.

**Table 2:** Weighted proportion of respondents purchasing at least one meal prepared outside home (either or not using OFDS) in the last 7 days across countries between 2018 and 2021, International Food Policy Study (N = 88337).

	2018 (n = 21986)		2019 (n = 20211)		2020 (n = 20905)		2021 (n = 20235)	
	%	95 % CI*	%	95 % CI	%	95 % CI	%	95 % CI
Overall**	80.4	79.7, 80.9	82.2	81.6, 82.8	73.2	72.5, 73.9	76.3	76.3,77.1
Australia	77.5	76.1, 78.9	77.9	76.5, 79.3	75.7	74.3, 77.1	74.9	73.4, 76.4
Canada	76.8	75.1, 78.3	79.2	77.6, 80.6	68.9	67.3, 70.6	70.6	68.8, 72.3
Mexico	94.3	93.2, 95.2	94.9	94.1, 95.8	86.5	85.0, 87.8	90.1	88.4, 91.6
UK	71.5	70.0, 72.9	73.6	71.9, 75.1	56.3	54.7, 58.0	66.7	64.9, 68.3
US	84.2	82.7, 85.5	84.8	83.4, 86.1	78.1	76.6, 79.5	81.7	80.2, 83. 1

*95% confidence intervals; **all countries combined.

**Table 3:** Multiple binomial logistic regression model showing changes in purchasing at least one meal outside home (either or not using OFDS), in the last 7 days between 2018 and 2021, International Food Policy Study (N = 88337).

	Meal purchased outside home (either or not using OFDS)^					
YEAR	OR^a^	95 %CI^b^	p- value	AOR^c^	95 %CI^b^	p- value
Overall*						
2018	ref					
2019	1.13	1.06, 1.19	<0.001	1.09	1.02, 1.16	<0.001
2020	0.66	0.63, 0.71		0.61	0.57 0.64	
2021	0.78	0.74, 0.84		0.78	0.73, 0.84	

**Australia**						
2018	ref					
2019	1.02	0.91, 1.15	0.002	1.03	0.91, 1.16	0.003
2020	0.90	0.81, 1.01		0.91	0.81, 1.02	
2021	0.86	0.77, 0.97		0.86	0.76, 0.97	

**Canada**						
2018	ref					
2019	1.15	1.01, 1.30	<0.001	1.16	1.02, 1.32	<0.001
2020	0.67	0.59, 0.75		0.65	0.57, 0.73	
2021	0.73	0.64, 0.82		0.72	0.63, 0.82	

**Mexico**						
2018	ref					
2019	1.15	0.89, 1.49	<0.001	1.17	0.90, 1.52	<0.001
2020	0.39	0.31, 0.48		0.40	0.32, 0.50	
2021	0.56	0.43, 0.72		0.66	0.51, 0.86	

**UK**						
2018	ref					
2019	1.11	0.99, 1.24	<0.001	1.12	0.99, 1.26	<0.001
2020	0.52	0.47, 0.56		0.47	0.42, 0.52	
2021	0.79	0.72, 0.88		0.77	0.69, 0.86	

**US**						
2018	ref					
2019	1.05	0.91, 1.21	<0.001	1.04	0.89, 1.21	<0.001
2020	0.67	0.59, 0.77		0.61	0.53, 0.71	
2021	0.84	0.73, 0.97		0.86	0.74, 0.99	

^compared to a meal prepared at home (reference category); *all countries combined; a—Odds ratio; b—95% confidence intervals; c—Adjusted Odds ratio (covariates adjusted include age, sex, education, ethnicity and living with children).

### 3.2 Annual proportion of the respondents purchasing meals using OFDS in the past 7 days

Overall, across respondents in all countries between 2018 and 2021, the annual proportion of the respondents purchasing meals using OFDS in the past 7 days increased from 19 % to 25 % (Table 4). The proportion of respondents using OFDS between 2018 and 2021 increased among respondents in Australia, Mexico, and UK, and nearly doubled in Canada from 2018 to 2020 followed by a slight decline between 2020 and 2021. The proportion of respondents in the US using OFDS also increased between 2018 and 2020 but slightly declined in 2021. In the multiple multinomial regression model, adjusted country-specific models for Australia, Canada, and the US, compared to 2018, higher odds of purchasing meals using OFDS were observed from 2019 to 2021. In the adjusted all-country combined models and in Mexico and the UK separately, compared to 2018, higher odds of purchasing meals using OFDS were observed over time except for 2020 where lower odds of purchasing meals using OFDS were observed (Table 5). These trends were statistically significant (p < 0.001) in all countries combined and country-specific unadjusted models. However, in the adjusted models, these trends were statistically significant only for all countries combined and Australia.

**Table 4:** Weighted proportion of respondents purchasing at least one meal using OFDS in the last 7 days across countries between 2018 and 2021, International Food Policy Study (N = 88337).

	2018 (n = 21986)		2019 (n = 20211)		2020 (n = 20905)		2021 (n = 20235)	
	%	95 % CI*	%	95 % CI	%	95 % CI	%	95 % CI
Overall**	18.5	17.9, 19.1	19.9	19.2, 20.5	26.5	25.8, 27.2	24.9	24.2, 25.7
Australia	17.5	16.1, 18.9	17.5	16.2, 18.9	22.7	21.3, 24.1	25.5	23.9, 27.1
Canada	11.7	10.6, 12.9	13.5	12.3, 14.8	19.9	18.5, 21.4	19.2	17.8, 20.8
Mexico	27.9	26.3, 29.5	30.5	28.9, 32.1	37.5	25.8, 39.3	38.1	35.7, 40.4
UK	19.1	17.8, 20.4	19.4	17.9, 20.9	25.8	24.3, 27.3	27.9	26.3, 29.6
US	16.6	15.2, 17.9	17.8	16.4, 19.2	26.5	25.0, 28.1	21.1	19.6, 22.7

*95% confidence intervals; **all countries combined.

**Table 5:** Multiple multinomial regression model showing changes in purchasing at least one meal outside home not using OFDS and purchasing at least one meal outside home using OFDS in the last 7 days between 2018 and 2021, International Food Policy Study (N = 88337).

	Meal purchased outside home not using OFDS^						Meal purchased outside home using OFDS^					
YEAR	OR^a^	95 %CI^b^	p- value	AOR^c^	95 %CI^b^	p- value	OR^a^	95 %CI^b^	p- value	AOR^c^	95 %CI^b^	p- value
Overall*												
2018	ref						ref					
2019	1.11	1.05, 1.18	<0.001	1.08	1.01, 1.14	<0.001	1.18	1.10, 1.27	0.067	1.16	1.07, 1.26	<0.001
2020	0.55	0.52, 0.59		0.53	0.49, 0.56		1.05	0.98, 1.12		1.06	0.98, 1.14	
2021	0.69	0.65, 0.73		0.69	0.65, 0.74		1.12	1.04, 1.20		1.37	1.26, 1.48	

**Australia**												
2018	ref						ref					
2019	1.03	0.91, 1.16	<0.001	1.03	0.91, 1.16	<0.001	1.02	0.87, 1.19	<0.001	1.04	0.86, 1.24	<0.001
2020	0.82	0.73, 0.92		0.85	0.75, 0.95		1.20	1.03, 1.39		1.30	1.09, 1.54	
2021	0.74	0.65, 0.83		0.77	0.67, 0.87		1.30	1.12, 1.52		1.48	1.24, 1.77	

**Canada**												
2018	ref						ref					
2019	1.12	0.98, 1.28	<0.001	1.14	1.00, 1.30	<0.001	1.28	1.06, 1.54	0.008	1.35	1.11, 1.65	0.001
2020	0.56	0.49, 0.63		0.58	0.51, 0.65		1.26	1.07, 1.49		1.36	1.14, 1.64	
2021	0.62	0.55, 0.71		0.65	0.57, 0.74		1.29	1.09, 1.54		1.42	1.77, 1.72	

**Mexico**												
2018	ref						ref					
2019	1.11	0.85, 1.43	<0.001	1.13	0.87, 1.47	<0.001	1.25	0.95, 1.63	<0.001	1.29	0.99, 1.71	0.327
2020	0.31	0.25, 0.39		0.33	0.27, 0.41		0.57	0.45, 0.72		0.65	0.52, 0.83	
2021	0.46	0.39, 0.59		0.52	0.39, 0.68		0.79	0.61, 1.05		1.21	0.92, 1.59	

**UK**												
2018	ref						ref					
2019	1.12	0.99, 1.25	<0.001	1.12	0.99, 1.26	<0.001	1.09	0.94, 1.27	0.039	1.08	0.91, 1.28	0.002
2020	0.38	0.34, 0.42		0.38	0.34, 0.42		0.88	0.78, 1.00		0.89	0.77, 1.04	
2021	0.63	0.56, 0.71		0.65	0.58, 0.73		1.25	1.09, 1.43		1.39	1.19, 1.64	

**US**												
2018	ref						ref					
2019	1.02	0.89, 1.19	<0.001	1.03	0.88, 1.19	<0.001	1.12	0.93, 1.34	0.303	1.14	0.93, 1.39	0.006
2020	0.55	0.48, 0.63		0.54	0.47, 0.52		1.16	0.98, 1.36		1.15	0.96, 1.39	
2021	0.78	0.67, 0.89		0.8	0.69, 0.93		1.11	0.93, 1.32		1.31	1.08. 1.61	

^compared to a meal prepared at home (reference category); *all countries combined; a—Odds ratio; b—95% confidence intervals; c—AOR: Adjusted Odds ratio (covariates adjusted include age, sex, education, ethnicity and living with children).

Supplementary Table 1a–c shows the proportion of respondents using different purchase and delivery modes among those purchasing food prepared outside home in the last 7 days by country and year. The proportion of respondents ordering directly from a restaurant declined over time while the proportion of respondents ordering in person at a restaurant/food outlet within or more than 5 minutes of home (excluding delivery) fluctuated between 2018 and 2021 but overall, declined over time. Similar patterns were observed across countries over time, where purchasing meals prepared outside home across all purchasing formats generally declined over time, except for OFDS use which increased in all countries overtime.

### 3.3 Annual average number of meals per week purchased using OFDS and other purchase formats

**Fig. 1:**
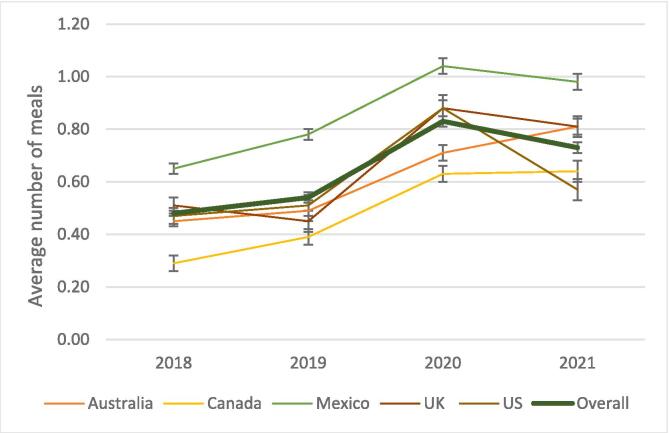
Weighted average number of meals per week prepared outside home using OFDS in the last 7 days across countries between 2018 and 2021, International Food Policy Study (N = 88337).

Fig. 1 shows the average number of meals purchased using OFDS nearly doubled from 0.4 meals in 2018 to 0.8 meals in 2020 and slightly declined to 0.7 meals in 2021. For other purchasing formats, there was a decline over time with some fluctuation in the average number of meals purchased in-person at a restaurant/food outlet within 5 minutes of home (excluding delivery) (Supplementary Fig. 1a–c). As shown in Fig. 1, some variations were observed across countries. For example, in the US, the average number of meals purchased using OFDS almost doubled between 2018 (0.5, SD = 0.03) and 2020 (0.9, SD = 0.05) and then reduced to 0.6 (SD = 0.03) meals in 2021. However, even with the 2021 decrease, the average number still increased by 1.3 times over the 4-year time span. For all years, the average number of meals purchased using OFDS was higher in Mexico than in Australia, Canada, the UK, and the US. Additionally, both the overall proportions of respondents and the average number of meals ordered directly from a restaurant including delivery and purchasing food in person at a restaurant/food outlet within or more than 5 minutes of their home (excluding delivery) decreased from 2018 to 2021 with some variations in 2021 (Supplementary Fig. 1a–c). Supplementary Table 2 shows the overall weighted average number of meals purchased outside home per week between 2018 and 2021 for all countries, which marginally declined from 3.1 to 2.8 meals, except in 2020 when it dropped to 2.6 meals.

## 4 Discussion

### 4.1 Summary of Key Findings

This study presents empirical evidence on the trends over time in purchases of all meals prepared outside home and those purchased using an OFDS in five upper middle- and high-income countries. Our study found that more than three quarters of the study sample across Australia, Canada, Mexico, the UK, and the US purchased at least one meal prepared outside home in the last week. In particular, the overall proportion of respondents purchasing meals using OFDS in the last 7 days increased over time with substantial changes particularly observed in 2020 and sustained in 2021 across all countries. Over the four-year period, the average number of meals purchased using OFDS increased approximately two-fold in all countries with little or no change to the overall purchasing of meals prepared outside home.

Our study substantiates emerging evidence (Morgan, 2020, Keeble et al., 2020) indicating increase in OFDS use over time. Though relatively small, some variations were observed in OFDS use in 2020 across countries. These observed differences in OFDS use were likely due to a combination of factors, including diversity in food culture, differences in OFDS market penetration and geographical remoteness that influence the access and availability of food outlets on OFDS, as well as the way in which people interact with OFDS (Statistica, 2023, Keeble et al., 2021, Chowdhury, 2023). Our study found that respondents from Mexico had the highest percentage of meals purchased outside home and using OFDS, followed by respondents from the UK, Australia, the US, and Canada. Alongside, an overall downward trend over time was observed for purchasing meals prepared outside home directly from a restaurant (including delivery) or in person at a restaurant/food outlet within or more than 5 min of your home (excluding delivery) across all countries. Our results suggests that popularity of OFDS is rising and is substituting conventional ways of purchasing food prepared outside home, to some extent.

The apparent increase in consumer preference for using OFDS over other traditional forms of purchasing food may have been linked to the COVID-19 pandemic (Chew and Lopez, 2021). Measures such as lockdown conditions and social distancing during the COVID-19 pandemic outbreak in 2020 led to the shutdown of restaurants across the world (Chew and Lopez, 2021). This likely provided impetus for OFDS to evolve and expand. While reports from Uber EATS® estimated that in 2021 consumers from 17 countries spent three times more on OFDS compared to 2020 (American Journal of Transportation, 2021), the findings from our study showed slow growth in the proportion of respondents using OFDS from 2020 to 2021. While the trends we observed may reflect a period of transition after the early stages of the COVID-19 pandemic for both food service owners and consumers, this slow growth may suggest a potential shift towards a more gradual increase in OFDS usage in subsequent years, aligning with broader trends in online shopping, including the online grocery shopping (Tyrväinen and Karjaluoto, 2022). It will be important to continue to monitor OFDS use, and the healthiness of food purchased on OFDS over time.

Research from multiple countries shows that OFDS largely offer food that is energy-dense and nutrient-poor (Brar and Minaker, 2021; Partridge et al., 2020). Policies aimed at OFDS such as mandated nutrition information for menu items could be extended to the restaurants or fast-food franchises more broadly to monitor the entire food-away-from-home sector in general. In addition to increasing accessibility of unhealthy foods (Partridge et al., 2020), research also suggests that OFDS have increased unsafe working conditions for food delivery workers (Mcdonald et al., 2019) and waste generated from food packaging (Gallego-Schmid and Azapagic, 2019, Song et al., 2018), thus, posing a threat to both health and climate. Hence, future research is needed to a) develop standardised tools to monitor multiple elements beyond the retail food environment to assist in evaluating the health and climate benefits of OFDS and b) identify ways to provide a better understanding of how these services influence the purchase and consumption of unhealthy foods, in real-time. This research may further inform the policy actions needed to improve the healthiness of the food purchased on OFDS. Research suggests that OFDS largely promote unhealthy food offerings that are often targeted and personalised to certain population groups such as younger age groups through aggressive marketing (Horta et al., 2022). Future research is required to investigate the types of marketing strategies employed by OFDS and how they differ based on the healthiness of the food product, consumer preference, demographics, and socioeconomic status. This will be useful to identify the extent and nature of unhealthy food marketing to at-risk population groups with an increased likelihood of poor health outcomes and demonstrate the need for policy actions to regulate the OFDS for public health benefit.

This study showed that a high proportion (on average >=1 meals per week) of meals prepared outside home were purchased via other conventional food purchase formats including ordering food directly from a restaurant (including delivery) and purchasing in person at a restaurant/food outlet regardless of distance from home, across all countries. This indicates that while OFDS use is rising, access to the other traditional modes (Bates et al., 2023, Granheim et al., 2022) of food purchasing continue to remain relevant. These findings reemphasise the need to strengthen the existing public health nutrition strategies and policies to tackle unhealthy retail food environments (Kraak et al., 2017, Gupta et al., 2023, Gupta et al., 2021). Future exploration on how similar strategies can be applied to include online food retail environment is warranted. For example, within the OFDS apps, restricting unhealthy food and brand marketing in advertisements, push notifications and sign-posts; introducing recipe reformulation, and labelling to promote healthy food options (World Health Organisation, 2021, Vanderlee et al., 2023).

### 4.2 Strengths and limitations

Strengths of this study include four years of annual data collection with large sample sizes which allowed us to explore changes in purchases of meals prepared outside the home, and purchases using OFDS. The period assessed in the study spanned 2018 to 2021, permitting evaluation of changes in the use of OFDS associated with the onset of the COVID-19 pandemic. Nonetheless, there are some limitations common to survey research. First, measures of number of meals purchased outside home and using OFDS were self-reported and can be subject to social desirability and recall bias. Second, respondents were recruited using non-probability-based sampling; therefore, the findings do not necessarily provide nationally representative estimates, despite that, efforts to enhance the representativeness of the data included application of post-stratification weights. In particular, the data from Mexico had higher levels of education in the responding population than census estimates, particularly in 2018–2020, with weighting of the data unlikely to fully account for this bias.

## 5 Conclusion

Our study found that nearly three-quarters of the adult population across Australia, Canada, Mexico, the UK, and the US purchased at least one meal prepared outside home from 2018 to 2021, and one-quarter to one-third used an OFDS. While the results showed that, in general, the number of meals prepared outside home remained mostly stable over the four-year period, the average number of those meals ordered using OFDS increased over time. This indicates that OFDS may be substituting conventional forms of food purchasing. As many food outlets offer a diverse range of food options, it is imperative to monitor the trends in OFDS use, the healthiness of food items sold on OFDS, and consumption of foods purchased outside the home more generally. This will help inform policies to limit the potential for OFDS to exacerbate existing trends toward unhealthy diets.

## 6 Ethics Approval and Consent to Participate.

The IFPS was reviewed by and received ethics clearance through a University of Waterloo Research Ethics Committee (ORE#30829). Consent was obtained from the participants prior to commencing the survey, meeting the institutional guidelines for protection of human subjects concerning safety and privacy. Further ethics clearance for this secondary data analysis was obtained from the Deakin University Research Ethics Committee (DUHREC#2023–100).

Consent for Publication

Not applicable.

Availability of Data and Materials.

The datasets used and analysed during the current study are available on reasonable request to the data custodian (DH).

## Funding

Funding for the International Food Policy Study was provided by a Canadian Institutes of Health Research (CIHR) Project Grant (PJT-162167), with additional support from the National Institute of Diabetes and Digestive and Kidney Disorders of the National Institutes of Health (R01 DK128967), the Public Health Agency of Canada (PHAC), and a CIHR-PHAC Applied Public Health Chair. The content is solely the responsibility of the authors and does not necessarily represent the official views of the Canadian Institutes for Health Research, the National Institutes of Health, or other sources of funding. The funding agencies had no role in the design of the study, the collection, analysis, or interpretation of data, or in the writing or decision to submit the manuscript for publication.

The current study was supported by AG’s Deakin University Executive Dean’s Health Research Fellowship and Victorian Health Promotion Foundation Early Career Fellowship. AJC is supported by National Heart Foundation of Australia, grant number 102611. KB is supported by a Fellowship [106716] from the National Heart Foundation of Australia. GS is a recipient of a National Health and Medical Research Council (NHMRC) Emerging Leadership Fellowship (2021/GNT2008535). AP is supported by an NHMRC Investigator Grant. CGD is supported by Fundación Alfonso Martín Escudero Postdoctoral Research Fellowship. LV is supported by a Canada Research Chair in Healthy Food Policy. This work is also supported by the NHMRC Centre of Research Excellence in Food Retail Environments for Health (RE-FRESH), grant number APP1152968.

## CRediT authorship contribution statement

**Adyya Gupta:** Writing – review & editing, Writing – original draft, Visualization, Validation, Methodology, Investigation, Formal analysis, Data curation, Conceptualization. **Gary Sacks:** Writing – review & editing, Supervision, Methodology, Formal analysis. **Adrian J. Cameron:** Writing – review & editing, Supervision, Methodology. **Catherine E. Huggins:** Writing – review & editing, Supervision, Methodology, Conceptualization. **Anna Peeters:** Writing – review & editing, Supervision, Methodology, Conceptualization. **Kathryn Backholer:** Writing – review & editing, Supervision, Methodology, Conceptualization. **Lana Vanderlee:** Writing – review & editing, Methodology, Conceptualization. **Christine M. White:** Writing – review & editing, Methodology, Investigation, Formal analysis, Data curation, Conceptualization. **Tailane Scapin:** Writing – review & editing. **Clara Gomez-Donoso:** Writing – review & editing. **Rebecca Bennett:** Writing – review & editing. **Joel A Dubin:** Writing – review & editing, Methodology. **David Hammond:** Writing – review & editing, Supervision, Resources, Project administration, Methodology, Investigation, Funding acquisition, Formal analysis, Data curation, Conceptualization.

## Declaration of competing interest

The authors declare the following financial interests/personal relationships which may be considered as potential competing interests: DH has provided paid expert testimony on behalf of public health authorities in response to legal claims from the food and beverage industry. All remaining authors declare no competing interest.

## Data Availability

Data will be made available on request.
