# Supplementary material for: Use of online food delivery services among adults in five countries from the International Food Policy Study 2018–2021
Source: Prev Med Rep. 2024 May 22;43:102766. doi: 10.1016/j.pmedr.2024.102766 (PMC11152731; doi:10.1016/j.pmedr.2024.102766)
Supplement: Supplementary Data 1 [file mmc1.docx]

**SUPPLEMENTARY FILES**

**Supplementary Table 1a: Weighted proportion of respondents ordering directly from restaurant including delivery, by country and year, International Food Policy Study (N=88337)**

|  | 2018 (n=21986) | | 2019 (n=20211) | | 2020 (n=20905) | | 2021 (n=20235) | |
| --- | --- | --- | --- | --- | --- | --- | --- | --- |
|  | % | 95%CI* | % | 95%CI | % | 95%CI | % | 95%CI |
| Overall** | 25.3% | 24.6, 25.9 | 22.7% | 22.0, 23.4 | 22.2% | 21.6, 22.9 | 20.4% | 19.7, 21.1 |
| Australia | 16.2% | 14.8, 17.6 | 14.8% | 13.6, 16.2 | 16.3% | 15.1, 17.5 | 18.2% | 16.9, 19.6 |
| Canada | 16.9% | 15.6, 18.4 | 14.8% | 13.6, 16.1 | 14.6% | 13.4, 15.7 | 15.2% | 13.9, 16.6 |
| Mexico | 55.1% | 53.2, 56.9 | 45.9% | 44.1, 47.7 | 39.8% | 37.9,41.6 | 38.6% | 36.2, 40.9 |
| UK | 16.5% | 15.3, 17.7 | 14.8% | 13.5, 16.2 | 13.4% | 12.3, 14.5 | 17.2% | 15.8, 18.5 |
| US | 24.7% | 23.1, 26.3 | 21.9% | 20.5, 23.6 | 26.8% | 25.3, 28.4 | 19.9% | 18.4, 21.4 |

*95% confidence intervals; **all countries combined

**Supplementary Table 1b: Weighted proportion of respondents purchasing food in person at a restaurant/food outlet within 5 minutes of your home (excluding delivery), by country and year, International Food Policy Study (N=88337)**

|  | 2018 (n=21986) | | 2019 (n=20211) | | 2020 (n=20905) | | 2021 (n=20235) | |
| --- | --- | --- | --- | --- | --- | --- | --- | --- |
|  | % | 95%CI* | % | 95%CI | % | 95%CI | % | 95%CI |
| Overall** | 38.9% | 38.1, 39.6 | 42.4% | 41.6, 43.2 | 36.9% | 36.2, 37.7 | 38.2% | 37.4, 39.1 |
| Australia | 37.2% | 35.4, 38.9 | 38.2% | 36.5, 39.9 | 38.1% | 36.5, 39.7 | 37.7% | 36.1, 39.4 |
| Canada | 36.6% | 34.8, 38.4 | 39.9% | 38.2, 41.7 | 33.5% | 31.9, 35.2 | 35.7% | 33.9, 37.5 |
| Mexico | 50.5% | 48.6, 52.3 | 53.9% | 52.2, 55.8 | 44.9% | 43.1, 46.8 | 45.3% | 42.8, 47.7 |
| UK | 27.5% | 26.0, 28.9 | 29.0% | 27.3, 30.7 | 22.7% | 21.4, 24.2 | 26.6% | 25.0, 28.2 |
| US | 45.5% | 43.7, 47.3 | 50.1% | 48.3, 51.9 | 44.6% | 42.9, 46.4 | 44.8% | 43.0, 46.7 |

*95% confidence intervals; **all countries combined

**Supplementary Table 1c: Weighted proportion of respondents purchasing food in person at a restaurant/food outlet more than 5 minutes of your home (excluding delivery), by country and year, International Food Policy Study (N=88337)**

|  | 2018 (n=21986) | | 2019 (n=20211) | | 2020 (n=20905) | | 2021 (n=20235) | |
| --- | --- | --- | --- | --- | --- | --- | --- | --- |
|  | % | 95%CI* | % | 95%CI | % | 95%CI | % | 95%CI |
| Overall** | 47.5% | 46.7, 48.3 | 45.9% | 45.1, 46.7 | 32.1% | 31.3, 32.8 | 38.9% | 38.0, 39.7 |
| Australia | 49.6% | 47.8, 51.3 | 45.9% | 44.2, 47.7 | 38.9% | 37.4, 40.6 | 37.4% | 35.7, 39.0 |
| Canada | 45.5% | 43.7, 47.3 | 43.3% | 41.6, 45.1 | 29.6% | 28.1, 31.3 | 35.2% | 33.5, 36.9 |
| Mexico | 487.9% | 46.1, 49.8 | 48.3% | 46.5, 50.1 | 33.7% | 32.0, 35.5 | 39.3% | 36.9, 41.8 |
| UK | 43.9% | 42.4, 45.5 | 43.1% | 41.2, 44.9 | 20.8% | 19.6, 22.3 | 33.2% | 31.6, 34.9 |
| US | 51.1% | 49.2, 52.9 | 48.9% | 47.1, 50.7 | 36.6% | 34.9, 38.2 | 45.9% | 44.1, 47.7 |

*95% confidence intervals; **all countries combined

**Supplementary Table 2: Weighted average number of meals per week prepared outside home (either or not using OFDS) in the last 7 days across countries between 2018 to 2021, International Food Policy Study (N=88337)**

|  | 2018 (n=21986) | | 2019 (n=20211) | | 2020 (n=20905) | | 2021 (n=20235) | |
| --- | --- | --- | --- | --- | --- | --- | --- | --- |
|  | Mean (SD) | 95% CI* | Mean (SD) | 95% CI | Mean (SD) | 95% CI | Mean (SD) | 95% CI |
| Overall** | 3.1(0.03) | 3.0, 3.14 | 3.2 (0.03) | 3.1, 3.2 | 2.6 (0.03) | 2.5, 2.6 | 2.8 (0.03) | 2.7, 2.8 |
| Australia | 2.7 (0.06) | 2.6, 2.9 | 2.8 (0.06) | 2.7, 2.9 | 2.6 (0.06) | 2.5, 2.7 | 2.6 (0.06) | 2.5, 2.8 |
| Canada | 2.5 (0.05) | 2.4, 2.6 | 2.6 (0.06) | 2.5, 2.7 | 1.9 (0.05) | 1.9, 2.1 | 2.2 (0.06) | 2.1, 2.4 |
| Mexico | 4.8 (0.08) | 4.6, 4.9 | 4.8 (0.08) | 4.6, 4.9 | 3.6 (0.07) | 3.5, 3.8 | 3.9 (0.09) | 3.8, 4.2 |
| UK | 2.2 (0.05) | 2.1, 2.3 | 2.2 (0.05) | 2.1, 2.3 | 1.5 (0.04) | 1.5, 1.6 | 2.0 (0.05) | 1.9, 3.4 |
| US | 3.4 (0.07) | 3.3, 3.5 | 3.5 (0.07) | 3.4, 3.6 | 3.2 (0.07) | 3.0, 3.3 | 3.2 (0.07) | 3.1, 3.4 |

*95% confidence intervals; **all countries combined

**SUPPLEMENTARY FIGURES:**

**Figure 1a: Average number of meals purchased per week directly from a restaurant (including delivery) in the last 7 days across countries between 2018 to 2021, International Food Policy Study (N=88337)**

**Figure 1b: Average number of meals purchased per week in person at a restaurant/food outlet within 5 minutes of your home (excluding delivery) in the last 7 days across countries between 2018 to 2021, International Food Policy Study (N=88337)**

**Figure 1c: Average number of meals purchased per week in person at a restaurant/food outlet more than 5 minutes of your home (excluding delivery) in the last 7 days across countries between 2018 to 2021, International Food Policy Study (N=88337)**
